# Soluble Phosphatidylserine Binds to Two Sites on Human Factor IXa in a Ca^2+^ Dependent Fashion to Specifically Regulate Structure and Activity

**DOI:** 10.1371/journal.pone.0100006

**Published:** 2014-06-30

**Authors:** Rinku Majumder, Tilen Koklic, Tanusree Sengupta, Daud Cole, Rima Chattopadhyay, Subir Biswas, Dougald Monroe, Barry R. Lentz

**Affiliations:** 1 Department of Biochemistry & Biophysics, University of North Carolina at Chapel Hill, Chapel Hill, North Carolina, United States of America; 2 Department of Medicine, University of North Carolina at Chapel Hill, Chapel Hill, North Carolina, United States of America; BioScience Project, United States of America

## Abstract

Clinical studies have demonstrated a correlation between elevated levels of FIX and the risk of coronary heart disease, while reduced plasma FIX causes hemophilia B. FIXa interacts with FVIIIa in the presence of Ca^2+^ and phosphatidylserine (PS)-containing membranes to form a factor X-activating complex (Xase) that is key to propagation of the initiated blood coagulation process in human. We test the hypothesis that PS in these membranes up-regulates the catalytic activity of this essential enzyme. We used a soluble form of phosphatidylserine, 1, 2-dicaproyl-*sn*-glycero-3-phospho-L-serine (C6PS), as a tool to do so. C6PS and PS in membranes are reported to regulate the homologous FXa nearly identically. FIXa binds a molecule of C6PS at each of with two sites with such different affinities (∼100-fold) that these appear to be independent. A high affinity C6PS binding site (K_d_∼1.4 µM) regulates structure, whereas a low-affinity binding site (K_d_∼140 µM) regulates activity. Equilibrium dialysis experiments were analyzed globally with four other data sets (proteolytic and amidolytic activities, intrinsic fluorescence, ellipticity) to unequivocally demonstrate stoichiometries of one for both sites. Michaelis-Menten parameters for FIXa proteolytic activity were the same in the presence of C6PS or PS/PC membranes. We conclude that the PS molecule and not a membrane surface is the key regulator of both factors Xa and IXa. Despite some minor differences in the details of regulation of factors Xa and IXa, the similarities we found suggest that lipid regulation of these two proteases may be similar, a hypothesis that we continue to test.

## Introduction

Factor X (FX) activation during blood coagulation is carried out by two alternative enzyme complexes: the extrinsic tenase (Xase) complex formed by Factor VIIa (FVIIa) and tissue factor (TF) and the intrinsic tenase complex composed of Factor IXa (FIXa) and Factor VIIIa (FVIIIa) [1]. There is general consensus that physiological coagulation is initiated by extrinsic Xase, while its propagation occurs *via* intrinsic Xase [1], [2]. FIXa is an *M*_r_ 44000, two-chain, vitamin K-dependent serine protease that hydrolyzes the Arg^194^-Ile^195^ peptide bond in the FX molecule to form activated FX (FXa) [3]. Although this reaction can proceed slowly in solution, it is significantly accelerated in the presence of negatively charged phospholipid surfaces [4]. *In vivo*, these surfaces are mainly provided by activated platelets or plasma lipoproteins (in the case of atherosclerotic lesions) [5]. For *in vitro* studies of FX activation by intrinsic Xase, synthetic phospholipid vesicles [usually composed of phosphatidylserine (PS) and phosphatidylcholine (PC) in a 25∶75 molar ratio] are often used to substitute for physiological surfaces.

Factor X activation by the intrinsic Xase complex is the rate-limiting step for thrombin generation during tissue factor-dependent coagulation [6], [7]. Animal models suggest that targeting of the intrinsic Xase complex may improve the risk/benefit ratio of antithrombotic therapy. It is well known that micro-particles (MPs, small membrane-bound vesicles that derive from activated platelets or damaged endothelial/epithelial cell membranes) circulate in the peripheral blood and play active roles in thrombosis, inflammation, and vascular reactivity [8]. In several disease states characterized by inflammation and vascular dysfunction, MP subpopulations are elevated, correlate with clinical events, and may have important roles in pathogenesis. One of the mechanisms by which platelet-derived MPs elicit procoagulant activity is by exposure of PS on their surface. It is likely that PS exposure on MPs also accounts for their role in the immune response [9] These observations make it imperative to understand the mechanisms by which PS regulates factors IXa. This report aims to increase our understanding of this PS regulatory mechanism.

PS is a key regulator of the prothrombinase complex [10]–[13], as it binds to FXa and FVa, inducing conformational changes in each that regulate both the activity of FXa and the assembly of the prothrombinase complex [10]–[14]. FIXa shows both structural and sequence homology to FXa [15], making it reasonable to hypothesize that PS also regulates FIXa. To test this, we employed dicaproyl-phosphatidylserine (C6PS), which is soluble below its well-established critical micelle concentration and whose binding to FXa has been well characterized [12], [14], [16]. We used intrinsic fluorescence and circular dichroism (CD) to reveal different aspects of FIXa structure and found that a Ca^2+^-dependent tight binding site is responsible for C6PS-induced conformational changes. We also tested the ability of C6PS to alter FIXa activity and found that it caused a ∼45 fold increase in proteolytic activity and 50% decrease in amidolytic activity when bound in a Ca^2+^-dependent fashion to weak regulatory sites that are apparently independent of the tight sites. FIXa with C6PS bound to this weak regulatory site showed proteolytic activity with Michaelis Menten constants similar to those obtained with FIXa bound to PS-containing membranes. We tested several soluble lipids (C6PG, C6PE, C6PC, C6(D)PS, and C6PA) to assess lipid-specificity and found that only C6PS bound to FIXa with high affinity and only C6PS and C6PE elicited changes in activity, although acidic lipids (C6PG, C6(D)PS, and C6PA) bound with weak affinity to elicit changes in intrinsic fluorescence. Finally, we confirmed the existence of only two sites using equilibrium dialysis and analyzed data from all five types of binding experiments to obtain the binding constants and stoichiometries of both types of sites. The results support our hypothesis that PS regulates FIXa similar to how it regulates FXa, although additional work is required to reveal the details of this regulation.

## Materials and Methods

### Materials

1,2-dicaproyl-sn-glycero-3-phospho-L-serine (C6PS) and all other lipids were purchased from Avanti Polar Lipids Incorporated (Alabaster, AL). Lipid stock solutions in buffer were prepared by measuring aliquots of appropriate lipid stocks in chloroform, evaporating the chloroform under a stream of nitrogen, re-solubilizing the lipid in cyclohexane, and then lyophilizing frozen solutions overnight. The resulting dry powder was dispersed in buffer (20 mM Tris, 150 mM NaCl, pH-7.5) and vortexed thoroughly [16].

Human FIXa and FX were purchased from Haemotologic Technologies Incorporated (Essex Junction, VT). The activities of FIXa and FXa were determined using the synthetic chromogenic substrates Pefachrome FIXa3960 (Pefa-3960) and Pefachrome FXa (Pefa-5523) both of which were purchased from Centerchem Incorporated (Norwalk, CT).

### Methods

#### Human FIXa Amidolytic Activity Assay

The amidolytic activity of human factor FIXa in the presence of various concentrations of C6PS was determined using a synthetic substrate, Pefa-3960. Amidolytic activity was measured in buffer (20 mM Tris, 150 mM NaCl, 0.6% PEG, pH 7.4, and either 5 mM CaCl2 or 0 mM CaCl_2_ with 1 mM EDTA) containing human FIXa (200 nM), chromogenic substrate (500 µM), and varying concentrations of C6PS (0–700 µM). The time course of absorbance yielded the amidolytic activity of FIXa. A small volume (89 µL) of appropriate calcium buffer was first incubated in a 96 well microplate at 37°C. Human FIXa was next allowed to bind C6PS by adding both to the buffer in the well and equilibrated for 5 minutes at 37°C. Finally, 7 mL of 10 mM Pefa-3960 was added to each well to bring the total volume of each solution to 140 mL. The absorbance at 405 nm was recorded every twenty seconds for 30 minutes using a vertically photometric VersaMax tunable microplate reader (Molecular Devices) to obtain the initial rate of Pefa-3960 hydrolysis. Rates of synthetic substrate hydrolysis were reported as a percent of the rate observed in the absence of phospholipid.

#### Proteolytic Activity Assay

The calcium- and phosphatidylserine-dependence of the proteolytic activity of FIXa was followed by measuring the generation of factor Xa amidolytic activity against the synthetic substrate Pefa-5523 at varying concentrations of CaCl_2_ and C6PS. Just as Pefa-3960 is used for FIXa, Pefa-5523 is used for the active site of FXa. Proteolytic activity mixtures contained buffer (20 mM Tris, 150 mM NaCl, 0.6% PEG, pH 7.4, 1 mM EDTA with 0, 1, 1.5, 2, 2.5, 3, 4, 5, or 6 mM CaCl_2_), human FIXa (5 nM), human FX (300 nM), Pefa-5523 chromogenic substrate (1 mM), and varying concentrations of C6PS (0–700 µM). A small volume of appropriate calcium buffer was first equilibrated in a 96 well microplate at 37°C. Human FIXa was next allowed to bind the appropriate amount of C6PS, which was added to the well and equilibrated with FIXa at 37°C for 5 minutes. Finally, a premixed solution of FX and chromogenic substrate pre-equilibrated for 2 minutes at 37°C was added to each well to bring the total volume of each well to 140 µL. After 30 seconds of mixing, the absorbance at 405 nm was recorded every twenty seconds for one hour using a VersaMax tunable micro plate reader (Molecular Devices). The data were analyzed as described elsewhere [17], [18] to obtain the initial rate of FX activation by FIXa.

#### Circular Dichroism Spectroscopy of FIXa

Circular Dichroism spectra of FIXa at 25.9°C were obtained on an Applied Photophysics (Leatherhead Surrey, Great Britain) Pi-star 180 spectrophotometer using a 1 cm path length cuvette and a 1 nm bandwidth. The spectra were recorded from 195 to 260 nm at 0.2 nm intervals, and corrected for the background contribution of buffer. The sample volume was 400 µL contained in a buffer (0.8 mM Tris, 100 mM NaCl, pH 7.4), FIXa (1 µM), 5 mM CaCl_2_, with varying concentrations of C6PS [0–600 µM]). Controls with C6PS were subtracted from each spectra. Observations were plotted as the instrumental output of ellipticity *θ* (mdeg).

#### Analysis of C6PX Binding

Binding was accessed by recording the appropriate observable (proteolytic activity, amidolytic activity change, intrinsic fluorescence change, or CD ellipticity ratio (θ_222_/θ_208_) as a function of soluble lipid (C6PX) concentration over a broad range below the critical micelle concentration (CMC) of the soluble lipid under experimental conditions. The CMC was measured as described earlier [10], [11], [19], [20]. Apparent dissociation constants for binding of FIXa to C6PS were obtained by fitting the experimental data to a simple, single-site binding model (hyperbolic fit since [C6PX]_free_≂[C6PX]_tot_), while using the approximation that [C6PX]_free_≈[C6PX]_total_ [19].

#### Equilibrium Dialysis to Asses Stoichiometry of C6PS Binding to FIXa

Our indirect binding measurements strongly support the hypothesis that two types of singly occupied C6PS sites exist on FIXa. We used equilibrium dialysis measurements to test this hypothesis. Experiments were performed using 2.0-ml Teflon dialysis cells (Spectrum Medical, Los Angeles, CA) with the two cells separated by a 2 KDalton-molecular-weight-cut-off membrane. Both chambers contained equal amounts of C6PS, while only one contained FIXa. Since inorganic phosphate determinations have inherent uncertainties of about 1–2% (volumetric measurement error), we used the highest practical concentrations of FIXa (20, 25 µM). C6PS was added to both chambers at sufficient concentrations (230, 240 and 250 µM) to occupy greater than 70% of the sites on FIXa (*i.e.*, to approach saturation). Under these conditions, the difference in inorganic phosphorous concentration between chambers (D*P*) will approach 10% of the lipid concentration needed to achieve near saturation. This required that multiple measurements be made in order to achieve the precision required to estimate binding stoichiometries. The two chambers were allowed to equilibrate at room temperature for 24 h while being rotated horizontally at 20 rpm. The protein concentration gradient between the two halves of the cell causes a difference in the total phospholipid concentration between the two halves of the cell. The difference in phospholipid concentration between the two chambers (D*P*) was measured by assaying eight aliquots out of each chamber for total phosphate [12], [14]. Total phosphate content was similarly measured for buffer plus protein alone as a control. A total of six independent measurements were made in this way. It should be evident that the inherent sensitivity of these measurements constrained the range of protein and C6PS concentrations that could be examined, but the analysis outlined in File S1 allowed the stoichiometries for each class of sites to be determined by these measurements.

## Results

### Effect of Short Chain Soluble Lipids on FIXa Structure

**Figure 1:**
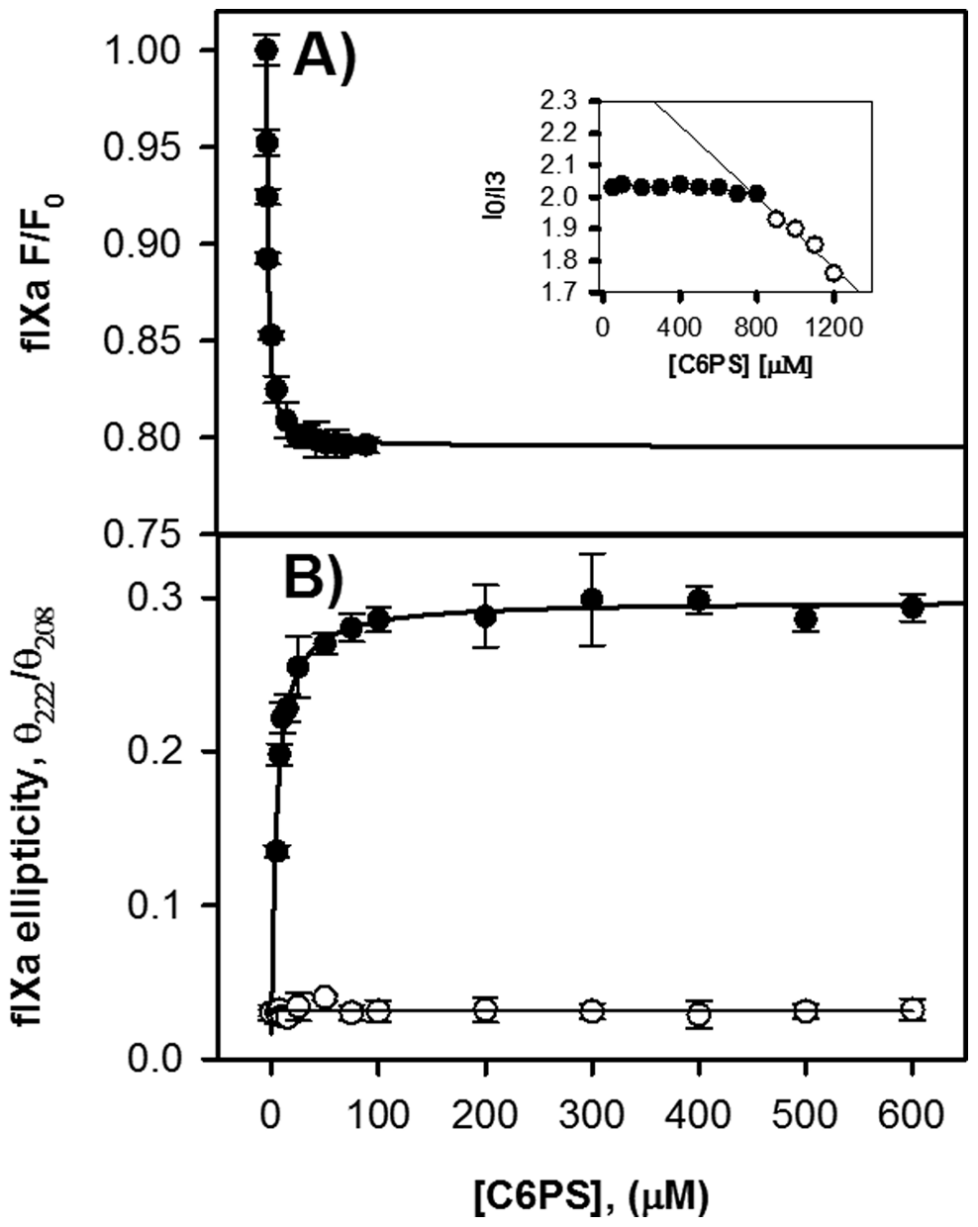
Structure Changes Induced in FIXa by C6PS as Detected by A: Intrinsic Fluorescence and B: Circular Dichroism Ratio θ_222_/θ_208_. Experiments in Frame A were performed with 0.20 µM FIXa in 50 mM Tris, 150 mM NaCl, 5 mM CaCl_2_, and 0.6% PEG, pH 7.5 at 22°C while those in Frame B were performed with 1 µM FIXa in in 150 mM NaCl, 5 mM CaCl_2_, and 0.6% PEG, with 0.8 mM Tris buffer at pH 7.4 and 26°C. Lines show analysis of the data according to simple single-site hyperbolic fits to yield apparent K_d_'s of 1.3 and 5.2 µM, respectively. The θ_222_/θ_208_ ratio obtained without Ca^2+^ is presented as open circles. The inset to figure 1A shows the fluorescence titration of pyrene in the presence of calcium and 0.20 µM factor IXa. There is a sudden decrease of fluorescence near a C6PS concentration of 950 µM. This decrease suggests that the CMC of C6PS occurs near this concentration.

We determined the intrinsic fluorescence of FIXa and the change in CD ellipticity ratio (θ_222_/θ_208_) of FIXa upon binding of C6PS in the presence of 5 mM Ca^2+^. These data are all presented in Figure 1. Each data set could be described using a simple, single-site binding isotherm (hyperbolic function) with effective dissociation constants: *K_d,eff_* = 1.3 µM for intrinsic fluorescence data (Figure 1A) and *K_d,eff_* = 5.2 µM for ellipticity ratio (Figure 1B). The similariy of these apparent Kd's indicates a common FIXa structural change in response to C6PS binding to a single site or to n_1_ identical and independent sites all with a common high affinity. This conclusion is supported directly by experimental data.

**Figure 2:**
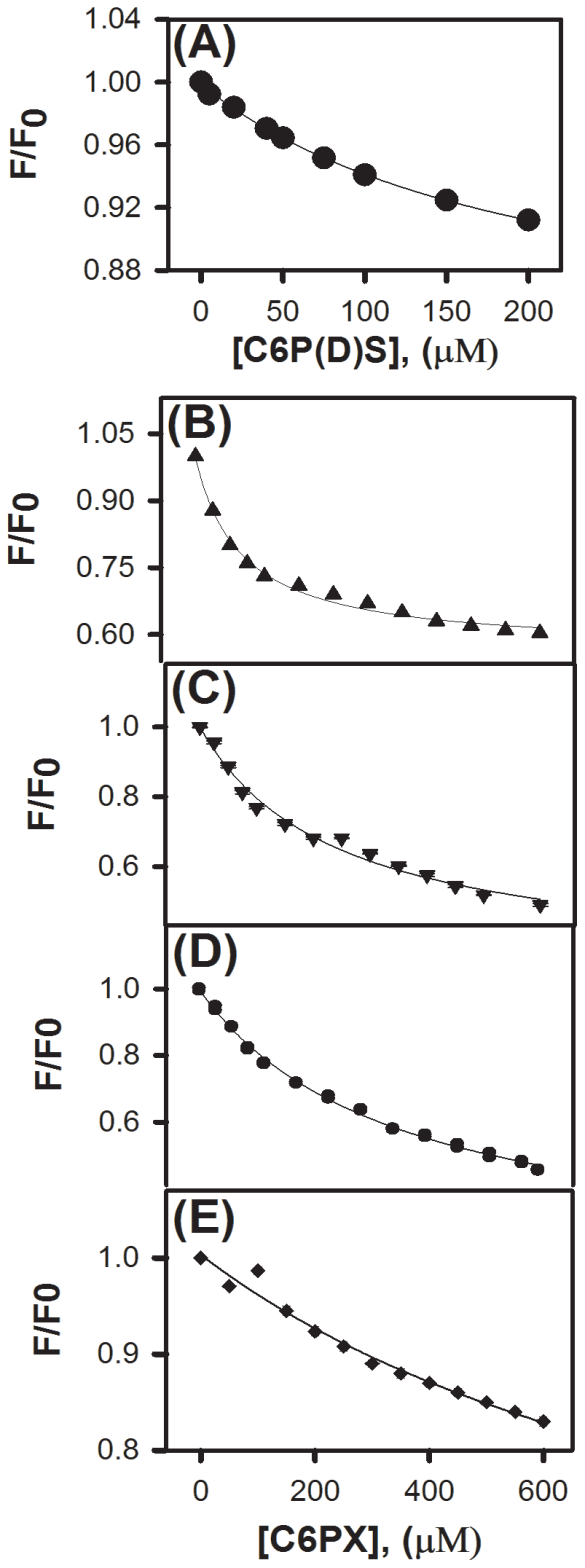
Binding of C6P(D)S, C6PE, C6PA, C6PG & C6PC to FIXa as Detected by Intrinsic Fluorescence. Integrated intrinsic fluorescence of 0.20 µM FIXa in 50 mM Tris, 150 mM NaCl, 5 mM CaCl_2_, and 0.6% PEG, pH 7.5 was measured as a function of C6P(D)S (Frame A), C6PE (Frame B), C6PG(Frame C), C6PA (Frame D) and C6PC (Frame E) concentration at 22°C to monitor binding. Single-site analysis (*i.e.*, hyperbolic fits) of these curves provided apparent K_d_'s of 208, 66, 238, 307 and 2000 µM, respectively.

Control experiments determined the critical micelle concentration (CMC) of C6PS [10] in the presence of 5 mM Ca^2+^ and 200 nM FIXa (inset to Figure 1A) in the presence of 0.6% PEG. These rely on changes in pyrene's complex florescence spectrum that is sensitive to the polarity of its environment. Pyrene is very hydrophobic and partitions strongly into the hydrophobic environment in the interior of a micelle. The ratio of the intensity of the pyrene emissions at 373 and 383 nm provides information about the polarity of the micro-environment of the probe, and, as such, is able to detect micelles with aggregation numbers as small as 10, as validated by a comparison with CMC determinations by surface tension and conductivity measurements [21], [22]. The CMC of C6PS under the conditions of our experiments was found to be 950 µM, well above the concentration used in our studies, thus confirming that the effects observed are due to C6PS binding to FIXa in a monomeric form. Binding of other short chain soluble lipids such as C6P(D)S, C6PE, C6PA, C6PG and C6PC to FIXa was also monitored by following changes in FIXa intrinsic fluorescence (Figure 2). These data were also adequately described by a simple, single-site binding model (hyperbolic function when [L]_tot_≈[L]_free_), yielding K_d_s of 208±18, 66±7, 238±42.7, 307±28.8, and 1000±45 µM for C6P(D)S, C6PE, C6PA, C6PG and C6PC, respectively (Table 1). These results show that only C6PS binds with high affinity to the site we have identified as influencing FIXa structure, while C6PE apparently binds to this site (*i.e.*, it also influences FIXa structure) but much more weakly than does C6PS

**Table 1:** Binding Affinity and Activity of FIXa in the Presence of Soluble Lipids.

Lipid	Kd, µM (determined by monitoring change in intrinsic fluorescence)	Intrinsic Fluorescence Change at Saturation	Kd, µM from Proteolytic Activity Increase	Proteolytic Activity increase (in fold) in presence of lipids	Kd, µM from Amidolytic Activity Change	Δ% amidolytic activity due to lipid binding at saturation
C6PS	1.3±0.2	−0.204	115±10	45 (3)	130±10	50 (3)
C6PE	66±7	−0.396	175±15	4(3)	250±12	5 (3)
C6PC	1000±45	−0.170	ND	None(2)	ND	None (2)
C6PG	307.0±28.8	−0.543	ND	None (3)	ND	None (2)
C6PA	238±42.7	−0.509	ND	None (3)	ND	None (3)
C6(D)PS	208±18	−0.088	ND	None (3)	ND	None (2)

None-no change.

#### Effect of soluble lipids on FIXa activity

**Figure 3:**
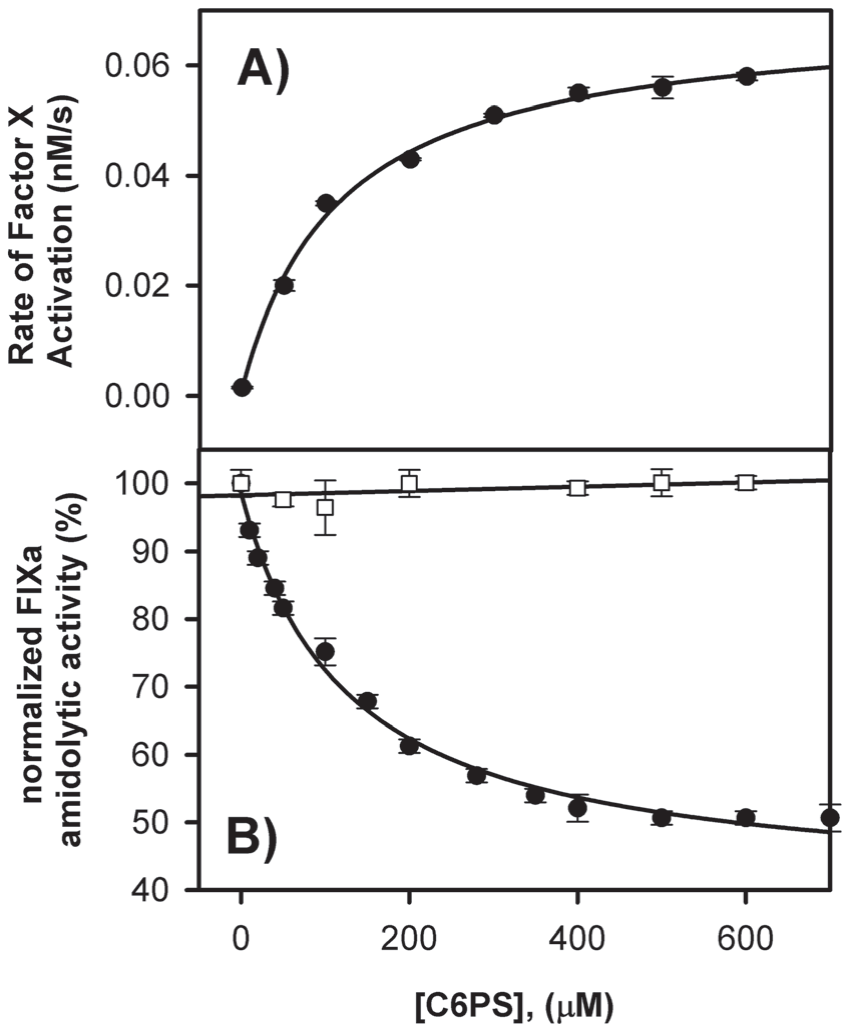
Variation of (A) Proteolytic and (B) Amidolytic Activities of FIXa Due to Addition of C6PS. **A:** Proteolytic activation of 200^2+^and 0.6%PEG, pH-7.5 at 37°C was monitored at different concentrations of C6PS, with a fit of the data to a single site (hyperbolic) model shown and yielding an apparent Kd of 115±10 µM **B:** Amidolytic activity of 300 nM FIXa in a buffer containing 50 mM Tris, 150 mM NaCl, 5 mM Ca^2+^and 0.6% polyethylene glycol was assayed using 1 mM synthetic substrate Pefa-3960 and increasing concentrations of C6PS, with a fit of the data to a single-site (hyperbolic) model, as shown, yielding an apparent K_d_ of 130±10 µM. The curve with open squares represents the amidolytic activity of FIXa in the absence of Ca^2+^ and with 1 mM EDTA.

Proteolytic activity of FIXa was measured by monitoring FIXa-catalyzed generation of FXa from FX in the presence of increasing concentrations of C6PS as described in Methods. Figure 3A shows the rate of FX activation at 5 mM Ca^2+^ with increasing C6PS concentration, with saturation still unachieved by almost 600 µM C6PS, well beyond the 30–50 µM seen for structural properties in Figures 1A and 1B. The titration curve was also well described by a single binding site hyperbolic model (Figure 3A inset), yielding a Kd of 115±10 µM for the interaction of FIXa with C6PS, with a ∼45-fold increase in proteolytic activity of FIXa in the presence of a saturating concentration of C6PS. Because the apparent Kd is nearly two orders of magnitude larger than that derived from C6PS-triggered structural changes, we conclude that proteolytic activity is regulated by a site different from the site revealed by structural measurements in Figure 1. With the exception of C6PE, other soluble lipids, such as C6PC, C6PG, C6PA, and C6DPS, had no effect on the proteolytic activity of FIXa (Table 1). C6PE increased the activity of FIXa by a factor of 4, a tenth of the 45-fold increase produced by C6PS (Table 1).

Figure 3B shows the amidolytic activity of FIXa (relative to activity in the absence of C6PS) as a function of C6PS concentration in the presence (filled circle, 5 mM) and absence of Ca^2+^ (open circles). Titration of FIXa by C6PS decreased its amidolytic activity by ∼50%. A single-site (hyperbolic) fit of the 5 mM Ca^2+^ data gave a Kd of 130±10 µM (Figure 3B inset), which was similar to that obtained from the proteolytic data in Figure 3A (115±10 µM), strongly implying that a common site (or set of *n_2_* equivalent sites) regulated both amidolytic and proteolytic activity and thus somehow the active site of FIXa. These low affinity sites are apparently different from those that produce global structural changes (Figure 1). Amidolytic activity was also followed in the presence of other soluble lipids. While C6PG, C6PC, C6PA and C6DPS did not alter the amidolytic of FIXa, a 5% decrease (as compared to the 50% decrease seen with C6PS) in amidolytic activity was observed with C6PE (Table 1). No change in amidolytic activity was observed in the absence of Ca^2+^ upon titration with C6PS (Figure 3B, opensquares).

### Effect of calcium on the structure and activity of FIXa in the presence of C6PS

**Figure 4:**
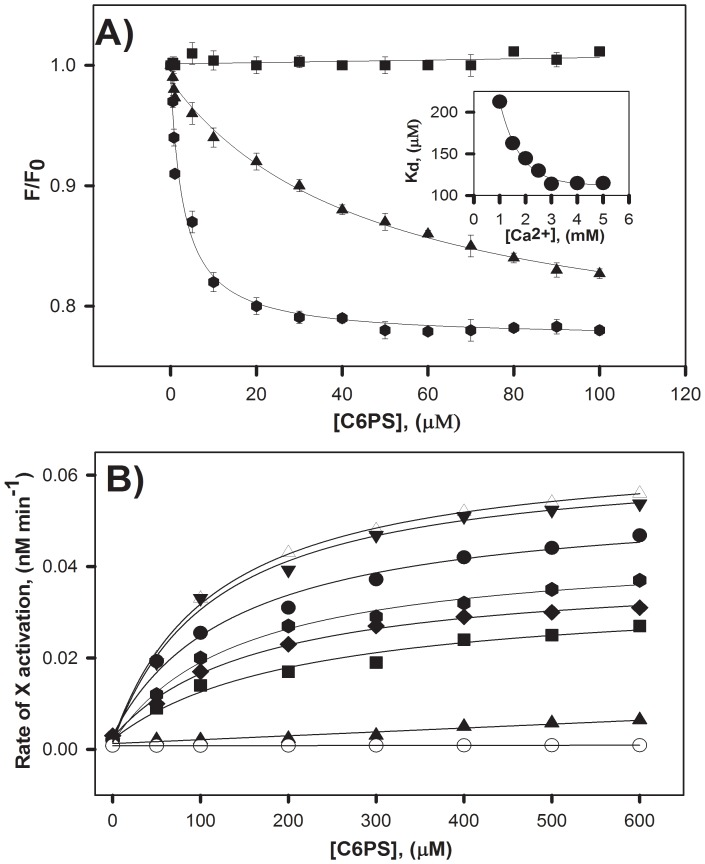
Effect of Ca^2+^ on the intrinsic fluorescence and proteolytic activity of FIXa in the presence of C6PS. **A.** The response of integrated intrinsic fluorescence to added C6PS is plotted for three different Ca^2+^ concentrations: 0 mM Ca^2+^ with 1 mM EDTA (squares), 1 mM Ca^2+^ (triangles), and 3 mM Ca^2+^ (hexagon). Hyperbolic fits of the data gave apparent K_d_'s∼47 µM and 3 µM for 1 and 3 mM Ca^2+^, respectively. **B.** The proteolytic activation of FX by 25 nM FIXa at pH-7.5, was monitored at 0 mM Ca^2+^ (with 1 mM EDTA) – open circle, 1 mM Ca^2+^ - triangle, 1.5 mM Ca^2+^ - square, 2 mM Ca^2+^ - diamond, 2.5 mM Ca^2+^ - hexagon, 3 mM Ca^2+^-circle, 4 mM Ca^2+^ - inverted triangle, and 5 mM Ca^2+^ - open triangle. The inset to 4A shows a plot of apparent K_d_'s obtained from hyperbolic fits of the activity data as a function of Ca^2+^ concentration. The plot shows 3 mM Ca^2+^ is sufficient for optimum FX activation by FIXa in the presence of C6PS.

In a fashion similar to that used in Figure 1A at 5 mM Ca^2+^, we monitored the change in intrinsic fluorescence of FIXa at 0, 1 and 3 mM Ca^2+^ to determine the optimum level of Ca^2+^ needed for FIXa binding to C6PS. Our result (Figure 4A) showed that C6PS binding increases in affinity with an increase in concentration of Ca^2+^.

We also monitored CD spectra of FIXa at different C6PS concentrations with 0 mM Ca^2+^ as described in Figure 1C (open circles), and the ratio of ellipticity at 222 nm to 208 nm remained unchanged with the addition of C6PS. Therefore, Ca^2+^ is required in order to observe the global change in FIXa structure induced by C6PS binding to its tight site on FIXa.

Measurement of proteolytic activity of FIXa with increasing concentrations of C6PS was also carried out at varying Ca^2+^ concentrations (Figure 4B). The apparent K_d_ of interaction of FIXa with C6PS was calculated at each Ca^2+^ concentration and plotted in the inset to Figure 4. From results in the absence of Ca^2+^ (open circle), it is evident that Ca^2+^ is absolutely essential for the FIXa proteolytic activity. The plot of apparent K_d_s *versus* Ca^2+^ concentration (Figure 4A inset) clearly shows that 3 mM Ca^2+^ was optimum for FX activation by FIXa in the presence of C6PS.

### Michaelis-Menten Kinetics of FX activation by FIXa in the presence of C6PS

**Figure 5:**
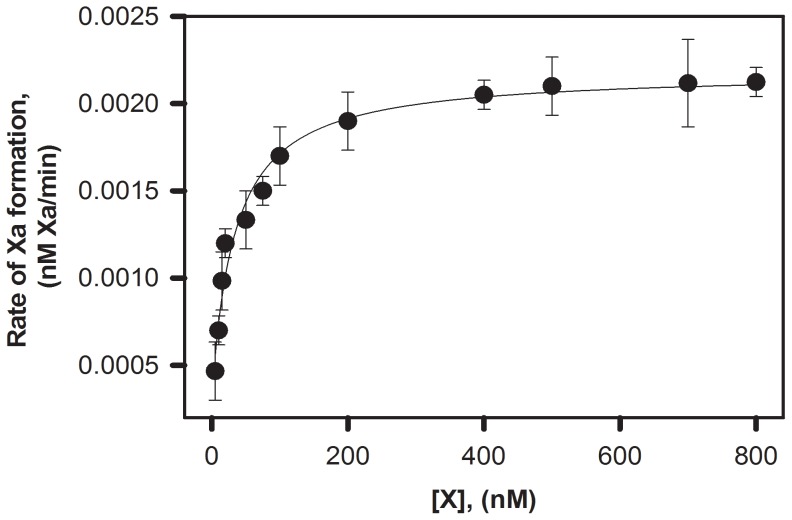
Michaelis-Menten kinetics of FX activation by FIXa in the presence of C6PS. The rate of activation of FX by FIXa in the presence of 400 µM C6PS, monitored at different concentrations of FX. Each test was performed in a buffer containing 50 mM Tris, 150 mM NaCl, and 5 mM Ca^2+^. The K_M_ and k_cat_ for this reaction are 33 nM and 0.00038/min.

Kinetic parameters k_cat_ and K_M_ of FX activation by FIXa were determined by monitoring the initial rate of FX activation in the presence of 400 µM C6PS at increasing concentration of FX (Figure 5). The values of k_cat_ and K_M_ obtained from fitting the curve were 0.00038±0.0002/min and 33±1 nM, respectively, yielding a k_cat_/K_M_ of 1.15×10^4^ M^−1^ min^−1^, similar to that obtained in the presence of PS/PC membranes (∼1.0×10^4^ M^−1^ min^−1^ obtained with 25/75 sonicated PS/PC vesicles) [17]. Thus, FIXa is comparably effective as an enzyme for FX activation either in the presence of PS/PC membrane or C6PS. To determine whether binding of PS to the tight binding site in FIXa (Kd∼2 µM) would cause any change in activity of FIXa, we repeated the experiment in Figure 5 with 20 µM C6PS so that only the tight binding site would be occupied. The k_cat_ and K_M_ were determined to be 0.00026±0.0001/min and 243±10 nM, respectively. The overall kcat/K_M_ (1.1±0.07×10^3^ M^−1^ min^−1^) at 20 µM C6PS is nearly the same as that obtained without any lipid (kcat/K_M_∼0.86×10^3^ M^−1^ min^−1^) [17]. This represents at best an ∼1.3-fold increase in k_cat_/K_M_ upon occupying the tight class of sites compared to a 13.4-fold increase upon occupying the loose class. We conclude that, while occupancy of the tight C6PS class of sites clearly alters FIXa structure, the structural changes provoked do not significantly alter FIXa activity.

### Equilibrium Dialysis

**Figure 6:**
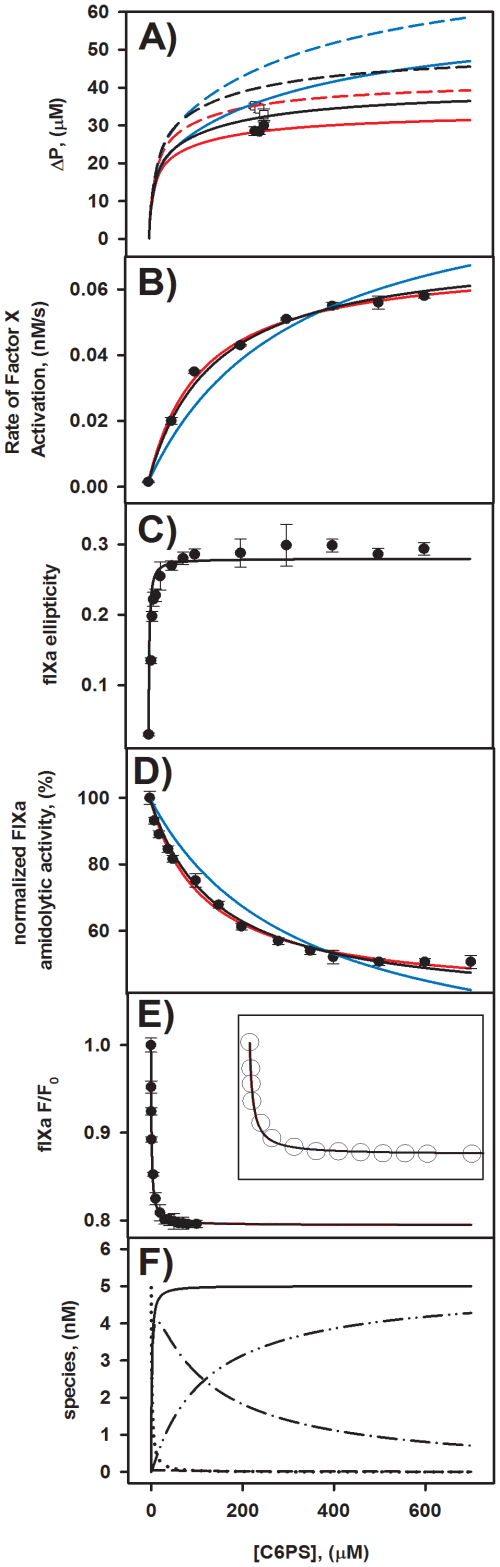
Simultaneous fit of functional and structural changes of FIXa versus concentration of soluble C6PS. Frame **A** plots results (ΔP as defined in File S1 *versus* C6PS concentration) of three independent equilibrium dialysis experiments performed at both 20 µM (closed circles) and 25 µM (open squares) of total FIXa. Frames **B** to **E** reproduce data from Figures 1 and 3, but with lines obtained as described in File S1. All data shown in Frames A to E of this figure were fitted globally by adjusting only *K_1_, K_2_, n_1_, and n_2_* (see File S1). The best fit for *n_1_* and *n_2_* as natural numbers was obtained for *n_1_, = n_2_* = 1 (Fit # 3) and is shown as solid black lines. Allowing *n_1_, and n_2_* to be real numbers (0.92 and 0.77, respectively) provided a fit that was statistically only slightly improved (; red lines. The next best fit (#6) was obtained with *n_1_* = 1 and *n_2_* = 2, but clearly was both numerically and visually (blue lines) much worse. Fits with other stoichiometries were even worse (Table 2). Frame **F** plots predicted FIXa species as calculated using parameters from Fit #3- (Table 2) and the model described in File S1 as a function of C6PS concentration.

Figures 1 and 3 provide clear evidence for two independent classes of sites with very different site binding constants. The curves drawn through these data are simple single site binding isotherms, although we do not know the actual stoichiometry of each class of sites. If the stoichiometries are other than 1, the actual K_d_'s for these sites will be larger than the apparent K_d_'s obtained from single site binding isotherms. In File S1, we derive the thermodynamic expressions for the equilibrium distribution of C6PS between sites assuming that each of the clearly identified independent classes of sites have stoichiometries *n_1_* and *n_2_* and site binding constants *K_1_* and *K_2_*. These *intensive* or global parameters should be the same for all experiments, while *extensive* parameters such as activities, molar ellipticities, and fluorescence changes associated with each species must be determined for each data set. We describe in File S1 how we approached the problem of fixing these parameters while globally fitting five data sets (intrinsic fluorescence, ellipticity ratio, proteolytic and amidolytic activities, and equilibrium dialysis). The equilibrium dialysis data are formally expressed in terms of the difference in ligand (*i.e.*, phosphate concentration) between the protein-containing and protein-free chambers of the dialysis apparatus (ΔP), as defined by Equation S14 in File S1. Six independently measured values for ΔP are plotted in Figure 6A *versus* the C6PS concentrations at which they were determined. These cluster into two groups of 3 that were obtained at two protein concentrations (20 and 25 µM;). ΔP divided by the total protein concentration was less than 2 for both equilibrium dialysis experiments. This suggests that the total occupancy of both sites (n_1_+n_2_) should be less than 2, but does not guarantee this, since the binding curves for occupancy of the weak site(s) (Figure 3) do not saturate. For this reason, we allowed n_1_+n2 to exceed 2 and considered (*n_1_,n_2_*) pairs during our data fitting (see File S1) of (0,0), (0,1), (1,0), (1,1), (0,2), (2,0) (1,2), (2,1), and (2,2). The best description of the data was obtained with *n_1_* = *n_2_* = 1, K_d1_ = 1.4±+1 uM, and K_d2_ = 142±6 µM (Table 2). The solid black lines through each data set in Figure 6 were obtained with the parameter values given in row 3 of Table 2, *i.e.*, (n_1_,n_2_) = (1,1). Figure 6F shows the species predicted by our model as a function of added C6PS using the global parameters *K*_d*1*_ = 1.4 µM, *K*_d*2*_ = 140 µM and *n_1_* = *n_2_* = 1.

**Table 2:** Results of globally fitting five data sets (FIXa proteolytic and amidolytic activity, FIXa intrinsic tryptophan fluorescence Intensity, and ellipticity from Figure 1 and 3, and equilibrium dialysis data) to the two-independent-site model with four global fitting parameters (best fit in bold).

Fit #	*n_1_*	*n_2_*	*n_1_*+*n_2_*		K_d,1_ (µM)	K_d,2_ (µM)	Proteolytic activity k_cat,3_ (10^−4^ s^−1^)	Relative amidolytic activity (%)
1	1	0	1	25	1.8	1060	325	0
2	0	1	1	17	220	308	500	173
3	**1**	**1**	**2**	**2.6**	**1.41**	**142**	**146**	**37**
4	2	0	2	26	1.9	1050	310	0
5	0	2	2	16	167	314	592	222
6	1	2	3	5.0	1.41	317	194	16
7	2	1	3	8.5	1.41	417	220	5
8	2	2	4	10.3	1.43	602	267	0
9	0.92	0.77	1.7	2.1	1.4	116	139	40

Fits 1–8 were obtained using the condition that that n_1_ and n_2_ must be natural numbers. A slightly lower was obtained if we relaxed this requirement (Fit #9). These fits as well as the next best fit (# 6) are compared to data in Figure 6 in the text.

## Discussion

### C6PS Binding Stoichiometry

As noted in File S1, a numerically better fit (*i.e.*, smaller) to all five data sets could be obtained if the constraint was removed that *n_1_*+*n_2_* must be whole numbers. This is shown as Fit #9 in Table 2 and illustrated by red lines in Figure 6. The lower for these parameters derives mainly from an improved description (dashed curves in Figure 6A) of equilibrium dialysis data (ΔP) collected at 25 µM FIXa (□ in Figure 6A). The physically unrealistic value of *n_2_* required to obtain this fit (0.77, Table 2, Fit #9) also requires a smaller K_d2_ (114 µM for Fit #9 *versus* 140 µM for Fit #3, Table 2) in order to correctly match the curvature of the activity titrations, which are the only titrations whose predicted shapes depend on *n_2_*. Despite the smaller, we must reject this set of parameters and focus on the parameters obtained with Fit #3. Table 2 and the blue lines in Figure 6 demonstrate that the next best whole number (*n_1_,n_2_*) pair (1,2; Fit #6 in Table 2) was unacceptable both statistically ( = 50) and visually. From this discussion, it is clear that our data and model support only the stoichiometry (1,1), *i.e.*, one tight and one weak site. The fact that a statistically improved fit can be obtained with the physically unreasonable value of *n_2_* = 0.77 implies that our model may be missing some aspect of the real system. This will be discussed below.

### Comparison to Prothrombinase Complex

A complex of FIXa with its cofactor FVIIIa (intrinsic Xase) activates FX by catalyzing the hydrolysis of a single peptide bond between Arg194 and Ile195 [23], [24]. This complex shows both structural and sequence homology to the FXa-FVa complex (prothrombinase) that catalyzes proteolysis of two peptide bonds in prothrombin to produce the central protease of blood coagulation, thrombin. It is well accepted that PS-containing membranes are required for optimal functioning of both these complexes and thus ultimately for thrombin generation. We know that C6PS regulates both the assembly and activity of the prothrombinase complex through interactions with C6PS-specific regulatory sites on both FXa and FVa [10], [12], [14], [16], [25]. C6PS binds to two sites on FXa. A tight (K_d_∼70–90 µM) C6PS binding site exits in the FXa EGF (epidermal growth-factor-like) domains [12], [14], [16] and regulates activity. These two domains can minimally bind C6PS but have optimal influence on activity when these domains are linked covalently to the Gla domain. Only acidic lipids, and mainly C6PS, can elicit structural or functional changes when bound to this site in the presence of Ca^2+^. A second, weaker (K_d_∼200–600 µM) binding site is located in the catalytic domain and is only minimally required for activity [12], [14]. The appearance of the weak site is linked to occupancy of the tight site and is suggested to be anomalous [14] and involve serine binding (minimal ligand is gyceryl-phosphoryl-serine) to a protein-binding site, either for FXa to form a FXa dimer [20], [25], or for FVa to form the prothrombinase complex [26]. Both sites require Ca^2+^ to optimally produce activity and structural changes.

While FIXa is structurally similar to FXa, we show here that its regulation by phosphatidylserine has both similarities to and differences from regulation of FXa by C6PS. First, we note three similarities revealed by our results:

1] As is the case for FXa [12], FIXa is capable of activating FX in the presence of soluble C6PS at a rate comparable to that observed with FIXa bound to a lipid membrane. We now know that, when bound to C6PS, the proteolytic activity of FIXa is increased 45-fold whereas amidolytic activity is decreased to 50% (40% for FXa) (Figure 3) (60-fold and for 40%, respectively for FXa [16]). The second order rate constant for proteolytic activation of prothrombin by membrane-bound FXa (9000 M^−1^ s^−1^) [25] is also very similar to that for FXa bound to C6PS in solution (14,000 M^−1^ s^−1^) [12]. Our data here show that the k_cat_ and K_M_ of FIXa-catalyzed activation of FX in the presence of 400 µM C6PS were 0.00038/min and 33 nM respectively (Figure 5) to give a k_cat_/K_M_ of 1.1×10^4^ M^−1^ min^−1^, similar to the rate constant found in the presence of PS/PC membranes (1.0×10^4^ M^−1^ min^−1^) [17]. So, just as for FXa, it is PS (either in solution or in membrane) not a membrane surface that regulates FIXa activity.
2] Regulation of both FXa and FIXa activity by C6PS requires Ca^2+^. Binding of C6PS to FXa is Ca^2+^ dependent, with optimal binding functional/structural responses reached by roughly 3 mM Ca^2+^ [16], [19]. When Ca^2+^ binds to FIX, the protein undergoes conformational changes [27]. The same appears to be true for FIXa, since Ca^2+^ is required for C6PS-induced FIXa conformation (Figure 1B) and activity (Figure 3B) changes as is the case for FXa [14]. Optimal binding of C6PS to FIXa occurs at around 3 mM Ca^2+^ (Figure 4A inset)), as is also the case for FXa. Thus, our results show that the same Ca^2+^ concentration dependence for C6PS regulation of FIXa as for C6PS regulation of FXa.
3] Lipid regulation of FIXa is specific for C6PS as is regulation of FXa [12]. This was confirmed by measuring the proteolytic and amidolytic activities of FIXa in the presence of C6(D)PS, C6PE, C6PA, C6PG and C6PC (Figure 2 and Table 1). None of these lipids had an effect on the catalytic efficiency of FIXa with the slight exception of C6PE. FXa binds C6PE with an affinity comparable to that with which it binds C6PS (∼70–90 µM for both lipids) [19]. Although the influence of C6PE on FXa activity is not addressed in the literature, it produced a 4-fold increase in proteolytic activity and a 5% decrease in amidolytic activity of FIXa, as compared to 45-fold increase and 50% decrease, respectively, for C6PS (Table 1).

Our results here deal solely with FIXa. We have yet to examine directly the influence of C6PS or C6PE on FVIIIIa and on its interaction with FIXa. However, C6PS binds to FXa [12] and FVa [13] to trigger formation of a fully active complex in solution in the absence of any membrane [10], so it would be instructive to explore the influence of C6PS and C6PE on FVIIIa and on its interaction with FIXa. C6PE is reported to bind to a recombinant human FVa_2_ (rHFVa_2_ – FVa_2_ is an isoform of FVa lacking a carbohydrate chain [11]) with an apparent Kd of ∼6 µM [19], while C6PS binds to bovine FVa with an apparent Kd of 20 µM [13]. No information was given about the stoichiometry of C6PE binding to rHFVa_2_, although the stoichiometry for C6PS binding to FVa is 4 [13], with a single site in the light chain C1 domain regulating rHFVa_2_'s ability to bind to FXa [25]. If we apply a simple equivalent, independent-site model to FVa binding to C6PS (apparent Kd∼20 µM), we would estimate that the regulatory site in the C1 domain might have a Kd of ∼5 µM, a prediction confirmed by studies of C6PS binding to rHFVa_2_ mutants (Kd∼3–4 µM [25]). Clearly, we need more detailed information (stoichiometries, site locations and affinities, *etc.*) about C6PS and C6PE binding to FVIIIa before we can fully understand the regulation of the intrinsic Xase by membrane lipids such as PS and PE and how this relates to regulation of the prothrombinase. These studies are beyond the scope of this report but are in progress.

Despite the similarities in PS regulation of factors Xa and IXa, there are also three apparent differences:

1] We show clearly that the two C6PS sites in FIXa behave as if they are independent, while the two C6PS sites on FXa appear to be linked [20]. However, this difference is less certain when we examine in more detail C6PS binding to the two proteins. First, the K_d_'s of the two C6PS sites on FXa differ by less than an order of magnitude (K_d1_∼70–90 µM and K_d,2_∼200–600 µM [12], [16], [20]), while those of sites on FIXa differ by two orders of magnitude (∼1.4 and 142 µM, Table 2). These are so different that it would be difficult to detect linkage if it existed, *i.e.*, site 1 would be essentially saturated before occupancy of site 2 became significant. Second, the apparent linkage between sites in FXa could be due to the fact that the apparent weak site in FXa is located in the catalytic domain near the active site [14] and near a residue shown to be included in the dimer interface. Since formation of a FXa dimer is triggered by occupancy of the tight, regulatory site [20], [25], the apparent linkage associated with C6PS binding to FXa could be due to structural changes associated with dimer formation. As yet, we do not know if C6PS binding triggers multimer formation by FIXa or whether there are functional consequences of multimer formation.
2] The activity-regulating C6PS binding site in FXa is the tight site (Kd∼70–90 µM) [12], [16], while in FIXa it is the weaker of two sites Kd∼140 µM). The tight C6PS binding site in FXa (Gla-EGFn domains) is near the membrane surface [14]), while we do not as yet know the location of either the tight or weak C6PS binding sites in FIXa. As discussed above, it is conceivable that the tight site in FIXa is near the membrane surface; if so, its occupancy could trigger conformational changes in the catalytic domain that allow formation of a FIXa-FVIIIa complex or a FIXa dimer, as is the case for the prothrombinase complex. Here again, locating the two C6PS sites in FIXa will be essential to fully unravel how PS regulates the intrinsic Xase and whether this is analogous to regulation of the prothrombinase. Efforts are under way to accomplish this using modified forms or fragments of FIXa.
3] Activation of prothrombin by FXa involves cutting two bonds, so it is difficult to make a clear comparison between the influences of C6PS on activities of these to proteases, but an analysis has been published of the influence of C6PS on the Michaelis-Menten parameters for conversion of the meizothrombin intermediate to thrombin [12]. Occupancy of the activity-regulating site on FIXa enhanced k_cat_/K_M_ by roughly 13-fold (Figure 5), while occupancy of the regulatory site on FXa increased k_cat_/K_M_ for meizothrombin activation by ∼200 fold [28]. Thus, C6PS had more than an order of magnitude greater influence on FXa proteolytic activity than it did on FIXa proteolytic activity. Binding of C6PS to the weak site of FIXa increased k_cat_ by only ∼1.5 fold, so most of the activity enhancement by C6PS was due to a reduction in K_M_ by ∼9-fold. Similarly, the influence of C6PS on the k_cat_/K_M_ of FXa was caused by a >26-fold decrease in K_M_ with an increase in k_cat_ of only ∼8 fold [28]. Thus, while the influence of C6PS on FIXa is less than its influence on FXa, it acts in both cases primarily by decreasing K_M_.

In summary, although there are apparent differences between the regulation of FXa and FIXa by lipids, it still may be that further studies will reveal the expected analogy. Given the key role of the intrinsic Xase in amplifying the initial response to injury mounted by the extrinsic Xase [29], understanding this lipid regulation is of great significance physiologically. Our results provide the groundwork needed to extend our knowledge of this regulation, but additional studies are needed to establish the locations of both the tight and weak sites on FIXa, determine whether C6PS triggers formation of a FIXa multimer as it does a FXa dimer, and finally to determine whether occupancy of the tight site is linked to binding of another protein such as a molecule of FIXa or the cofactor VIIIa.

## Supporting Information

File S1 **Supporting Information.**
(DOCX)

## References

[1] Mann KG (1999) Biochemistry and physiology of blood coagulation. Thromb Haemost 82: 165–174. 10605701

[2] Mann KG, Butenas S, Brummel K (2003) The dynamics of thrombin formation. Arterioscler Thromb Vasc Biol 23: 17–25. 12524220 10.1161/01.atv.0000046238.23903.fc

[3] Venkateswarlu D, Perera L, Darden T, Pedersen LG (2002) Structure and dynamics of zymogen human blood coagulation factor X. Biophys J 82: 1190–1206. 11867437 10.1016/S0006-3495(02)75476-3 PMC1301923

[4] van Dieijen G, Tans G, Rosing J, Hemker HC (1981) The role of phospholipid and factor VIIIa in the activation of bovine factor X. J Biol Chem 256: 3433–3442. 6782101

[5] Saenko EL, Shima M, Sarafanov AG (1999) Role of activation of the coagulation factor VIII in interaction with vWf, phospholipid, and functioning within the factor Xase complex. Trends Cardiovasc Med 9: 185–192. 10881749 10.1016/s1050-1738(00)00019-0

[6] Rand MD, Lock JB, van't Veer C, Gaffney DP, Mann KG (1996) Blood clotting in minimally altered whole blood. Blood 88: 3432–3445. 8896408

[7] Hockin MF, Jones KC, Everse SJ, Mann KG (2002) A model for the stoichiometric regulation of blood coagulation. J Biol Chem 277: 18322–18333. 11893748 10.1074/jbc.M201173200

[8] Ardoin SP, Shanahan JC, Pisetsky DS (2007) The role of microparticles in inflammation and thrombosis. Scand J Immunol 66: 159–165. 17635793 10.1111/j.1365-3083.2007.01984.x

[9] Rautou PE, Vion AC, Amabile N, Chironi G, Simon A, et al (2011) Microparticles, vascular function, and atherothrombosis. Circ Res 109: 593–606. 21852557 10.1161/CIRCRESAHA.110.233163

[10] Majumder R, Weinreb G, Lentz B (2005) Efficient thrombin generation requires molecular phosphatidylserine, not a membrane surface. Biochemistry 44: 16998–17006. 16363813 10.1021/bi051469f

[11] Majumder R, Weinreb G, Zhai X, Lentz BR (2002) Soluble phosphatidylserine triggers assembly in solution of a prothrombin-activating complex in the absence of a membrane surface. J Biol Chem 277: 29765–29773. 12045194 10.1074/jbc.M200893200

[12] Banerjee M, Drummond DC, Srivastava A, Daleke D, Lentz BR (2002) Specificity of soluble phospholipid binding sites on human factor Xa. Biochemistry 41: 7751–7762. 12056907 10.1021/bi020017p

[13] Zhai X, Srivastava A, Drummond DC, Daleke D, Lentz BR (2002) Phosphatidylserine binding alters the conformation and specifically enhances the cofactor activity of bovine factor Va. Biochemistry 41: 5675–5684. 11969429 10.1021/bi011844d

[14] Srivastava A, Wang J, Majumder R, Rezaie AR, Stenflo J, et al (2002) Localization of phosphatidylserine binding sites to structural domains of factor Xa. J Biol Chem 277: 1855–1863. 11707438 10.1074/jbc.M105697200

[15] Furie B, Bing DH, Feldmann RJ, Robison DJ, Burnier JP, et al (1982) Computer-generated models of blood coagulation factor Xa, factor IXa, and thrombin based upon structural homology with other serine proteases. The Journal of biological chemistry 257: 3875–3882. 7037788

[16] Koppaka V, Wang J, Banerjee M, Lentz BR (1996) Soluble phospholipids enhance factor Xa-catalyzed prothrombin activation in solution. Biochemistry 35: 7482–7491. 8652526 10.1021/bi952063d

[17] Rawala-Sheikh R, Ahmad SS, Ashby B, Walsh PN (1990) Kinetics of coagulation factor X activation by platelet-bound factor IXa. Biochemistry 29: 2606–2611. 2110473 10.1021/bi00462a025

[18] Rawala-Sheikh R, Ahmad SS, Monroe DM, Roberts HR, Walsh PN (1992) Role of gamma-carboxyglutamic acid residues in the binding of factor IXa to platelets and in factor-X activation. Blood 79: 398–405. 1730085

[19] Majumder R, Liang X, Quinn-Allen MA, Kane WH, Lentz BR (2011) Modulation of prothrombinase assembly and activity by phosphatidylethanolamine. The Journal of biological chemistry 286: 35535–35542. 21859710 10.1074/jbc.M111.260141 PMC3195639

[20] Majumder R, Wang J, Lentz BR (2003) Effects of Water Soluble Phosphotidylserine on Bovine Factor X(a): Functional and Structural Changes Plus Dimerization. Biophys J 84: 1238–1251. 12547804 10.1016/S0006-3495(03)74939-X PMC1302700

[21] Haque ME, Das AR, Moulik SP (1995) Behaviors of Sodium Deoxycholate (Nadc) and Polyoxyethylene Tert-Octylphenyl Ether (Triton X-100) at the Air/Water Interface and in the Bulk. Journal of Physical Chemistry 99: 14032–14038.

[22] Haque ME, Das AR, Moulik SP (1999) Mixed micelles of sodium deoxycholate and polyoxyethylene sorbitan monooleate (Tween 80). Journal of Colloid and Interface Science 217: 1–7. 10441405 10.1006/jcis.1999.6267

[23] Lawson JH, Mann KG (1991) Cooperative activation of human factor IX by the human extrinsic pathway of blood coagulation. J Biol Chem 266: 11317–11327. 2040636

[24] Fay PJ, Beattie TL, Regan LM, O'Brien LM, Kaufman RJ (1996) Model for the factor VIIIa-dependent decay of the intrinsic factor Xase. Role of subunit dissociation and factor IXa-catalyzed proteolysis. The Journal of biological chemistry 271: 6027–6032. 8626386 10.1074/jbc.271.11.6027

[25] Chattopadhyay R, Iacob R, Sen S, Majumder R, Tomer KB, et al (2009) Functional and structural characterization of factor Xa dimer in solution. Biophys J 96: 974–986. 19186135 10.1016/j.bpj.2008.10.013 PMC2716635

[26] Majumder R, Koklic T, Rezaie AR, Lentz BR (2013) Phosphatidylserine-induced factor Xa dimerization and binding to factor Va are competing processes in solution. Biochemistry 52: 143–151. 23214401 10.1021/bi301239z PMC3544317

[27] Jacobs M, Freedman SJ, Furie BC, Furie B (1994) Membrane binding properties of the factor IX gamma-carboxyglutamic acid-rich domain prepared by chemical synthesis. The Journal of biological chemistry 269: 25494–25501. 7929250

[28] Weinreb GE, Mukhopadhyay K, Majumder R, Lentz BR (2003) Cooperative Roles of Factor Va and Phosphatidylserine-containing Membranes as Cofactors in Prothrombin Activation. J Biol Chem 278: 5679–5684. 12438309 10.1074/jbc.M208423200

[29] Monroe DM, Hoffman M, Roberts HR (1996) Transmission of a procoagulant signal from tissue factor-bearing cells to platelets. Blood Coagulation & Fibrinolysis 7: 459–464. 8839998 10.1097/00001721-199606000-00005

