# Supplementary material for: Soluble Phosphatidylserine Binds to Two Sites on Human Factor IXa in a Ca2+ Dependent Fashion to Specifically Regulate Structure and Activity
Source: PLoS One. 2014 Jun 30;9(6):e100006. doi: 10.1371/journal.pone.0100006 (PMC4076177; doi:10.1371/journal.pone.0100006)
Supplement: File S1 — Supporting Information. (DOCX) [file pone.0100006.s001.docx]

**SUPPLEMENT to: Soluble Phosphatidylserine Binds to Two Sites on Human Factor IXa in a Ca^2+^ Dependent Fashion to Specifically Regulate Structure and Activity**

Rinku Majumder^1*^, Tilen Koklic^1#^, Tanusree Sengupta^1#^, Daud Cole^1^, Rima Chattopadhyay^1^, Subir Biswas^1^, Dougald Monroe^2^ & Barry R. Lentz^1^.

**THE THERMODYNAMIC MODEL**

In the two-independent-site model, we assumed that (n_1_ + n_2_) C_6_PS molecules binds to two classes of FIXa sites. For each class of sites, the site dissociation constants are assumed to be identical within that class (K_d,1_ and K_d,2_) and the sites are assumed to be independent. The data in Figures 1, 3, and 4 support this model, although these data cannot establish both the site dissociation constants and stoichiometry of these two classes of sites when fit individually. However, a simultaneous fit of these four independent experimental data sets along with equilibrium dialysis data (Figure 6) can establish the dissociation constants and stoichiometries. The stoichiometries of C6PS binding to FIXa must be whole numbers, therefore we treated them as such in order to obtain the best fit. Because the sites are independent, only two binding constants are required to define and five species defined by occupation of these sites$FIXaPS_{n_{1}}^{1}$ $PS_{n2}^{2}FIXa$ $FIXaPS_{n_{1}}^{1}$ $PS_{n_{2}}^{2}FIXaPS_{n_{1}}^{1}$ $PS_{n2}^{2}FIXa$ $PS_{n_{2}}^{2}FIXaPS_{n_{1}}^{1}$:

S1

$$K_{d,1}=\frac{[FIXa][PS]}{[FIXaPS^{1}]}$$

S2

$$K_{d,2}=\frac{[IXa][PS]}{[PS_{n_{2}}^{2}IXa]}$$

The type of site being occupied is indicated by superscripts. These binding events can produce four classes of stoichiometric species that differ according to how many sites of each class are occupied: $FIXa;FIXaPS_{i}^{1};PS_{j}^{2}FIXa;PS_{j}^{2}FIXaPS_{i}^{1}$. Within each class, there exist several molecular species, *e.g.*, for $FIXaPS_{i}^{1}$, these are $C_{i}^{n_{1}}$such species; for $FIXaPS_{i}^{1}$, these are $FIXaPS_{1}^{1},FIXaPS_{2}^{1}...FIXaPS_{n_{1}}^{1}$. If only one class of sites exist, this becomes the classical Hill model in which species with multiple ligands bound ligands are entropically favored. The probabilities of observing these species (*i.e.*, their thermodynamic concentrations) are defined by a molecular partition function for a system open with respect to the numbers of C6PS molecules bound to each class of sites. This is formally termed a grand canonical partition function, $\vartheta$ for the macromolecule as a system open with respect to ligand {Hermans, 2014 #308}:

$\vartheta=1+\sum_{i=1}^{n_{1}} C_{i}^{n_{1}}\left(K_{1}[PS]\right)^{i}=\left(1+K_{1}[PS]\right)^{n_{1}}$ S3

, with the probability of observing any species with *i* bound C6PS being $P_{i}=\frac{C_{i}^{n_{1}}\left(K_{1}[PS]\right)^{i}}{\left(1+K_{1}[PS]\right)^{n_{1}}}$. S4

This can be easily generalized to the partition function for the model considered here:

$\vartheta=\left(1+K_{1}[PS]\right)^{n_{1}}\left(1+K_{2}[PS]\right)^{n_{2}}$ S5

In this case, the probability of observing any stoichiometric species with *i* ligands in type 1 sites and *j* ligands in type 2 sites is:

$P_{i,j}=\frac{C_{i}^{n_{1}}C_{j}^{n_{2}}\left(K_{1}[PS]\right)^{i}\left(K_{2}[PS]\right)^{j}}{\left(1+K_{1}[PS]\right)^{n_{1}}\left(1+K_{2}[PS]\right)^{n_{2}}}$ S6

, and the total probabilities of occupancy of the two classes of sites are:

. S7

In Equation S7, P_0_, P_1_ and P_2_ are the probabilities that neither class of sites are occupied and that the first or second classes of sites are occupied.

**COMPUTATIONAL PROCEDURES**

Now we make simplifying assumptions in order to write expressions for observables. We assume that any species with any number of C6PS in site 1 has properties associated with occupancy of that site alone and similarly for site 2. The intrinsic tryptophan fluorescence intensity (*F*), or ellipticity ratio (θ_222_/θ_208_), in the presence of soluble PS, can be written as:

$F=P_{0}\cdot F_{FIXa_{free}}+P_{1}\cdot F_{FIXaPS^{1}}+P_{2}\cdot F_{PS^{2}FIXa}+P_{1}\cdot P_{2}\cdot\Delta F_{PS^{2}FIXaPS^{1}}$ S8

, where $F_{FIXaPS^{1}}$ and $F_{FIXaPS^{2}}$ are intrinsic fluorescence intensities with the first and second class of sites occupied, respectively, and $\Delta F_{PS^{2}FIXaPS^{1}}=F_{PS^{2}FIXaPS^{1}}-F_{FIXaPS^{1}}-F_{PS^{2}FIXa}$ . The last term in Equation S8 appears only when the properties of the species $PS^{2}FIXaPS^{1}$ are not equal to the sum of contributions from species $FIXaPS^{1}$ and $PS^{2}FIXa$. In the case of our fluorescence measurements, we assume that ΔF = 0 and $F_{PS^{2}FIXa}=F_{FIXa_{free}}$, since there is no indication from experiments that weak site binding alters intrinsic fluorescence (Figure 1). A similar expression can be written for observed ellipticity in terms of the molar ellipticity ratios (*[θ_222_/θ_208_]_i_*) for species with sites of type 1 or 2 occupied.

$\frac{\Theta_{222}}{\Theta_{208}}=P_{0}\cdot+P_{1}\cdot\left(\frac{\theta_{222}}{\theta_{208}}\right)_{1}+P_{2}\cdot\left(\frac{\theta_{222}}{\theta_{208}}\right)_{2}+P_{1}\cdot P_{2}\cdot\Delta\left(\frac{\theta_{222}}{\theta_{208}}\right)_{1,2}$ S9

In order to describe proteolytic activity of FIXa against factor X (FX) in the presence of increasing concentration of C6PS, we used the classical form of the Michaelis-Menten equation:

$\frac{dP}{dt}=k_{cat}[E_{total}]\frac{[FX]}{K_{M}+[FX]}$. S10

At very large substrate concentrations ([FX*_total_*]>>K_M_ =33 nM), the Michaelis-Menten equation transforms into: $\frac{dP}{dt}\approx(k_{cat})[E_{total}]$ S11

, where [FX_total_] = 300 nM, and [E_total_] = [FIXa _total_] = 5 nM in our experiments. Since these conditions satisfy ([FX*_total_*]>>K_M_,, we can write observed activity as:

$R=\frac{dP}{dt}=[IXa]_{tot}\left(P_{0}\cdot k_{cat,0}+P_{1}\cdot k_{cat,1}+P_{2}\cdot k_{cat,2}+P_{1}\cdot P_{2}\cdot\Delta k_{cat,3}\right)$ S12

Note that, unlike for the intrinsic fluorescence, it is more difficult to be sure that binding to the tight site does not alter activity (k_cat,1_ = k_cat,0_) and to predict that Δk_cat,3_ = 0. Nonetheless, we found that a good description of our activity data (Figure 3) was obtained with these assumptions. An exactly analogous expression is written for the % amidolytic activity except that the extensive k_cat_ parameters are replaced by extensive %activity parameters *δ_i_*.

The same model was applied to fit equilibrium dialysis data. The fixed total concentrations of C6PS and FIXa provide normalizing conditions:

$[PS_{tot}]=[PS]+[IXa_{tot}]\left(P_{1}\cdot n_{1}+P_{2}\cdot n_{2}+P_{1}\cdot P_{2}(n_{1}+n_{2})\right)$. S13

$[FIXa_{tot}]-[FIXa_{free}]=[FIXa_{tot}]\cdot\left(P_{1}\cdot n_{1}+P_{2}\cdot n_{2}\right)$ or $P_{0}+P_{1}+P_{2}=1$

In Equation S13, the probabilities depend on the concentration of ligand, on K_d_,*_i_*, and on n*_i_* (see Equa. S7). Even when determined by global fitting of fluorescence, CD, and activity datasets, values of the parameters K_d_,*_i_*, and n*_i_* are linked. However, equilibrium dialysis data allow n_1_ and n_2_ to be uniquely fixed (see Table 2). The quantity ΔP is the difference between total and free C6PS. It is measured by equilibrium dialysis as the difference in ligand concentration between two compartments, one containing the macromolecule (FIXa) plus free ligand and one containing only free ligand (C6PS):

$\Delta P=[PS]_{tot}-[PS]=[IXa_{tot}]\left(P_{1}\cdot n_{1}+P_{2}\cdot n_{2}+P_{1}\cdot P_{2}(n_{1}+n_{2})\right)$. S14

Recognizing that *n_1_* and *n_2_* can be only whole numbers (*i.e.*, 0, 1, 2, *etc.*) can lead to a simplification during data fitting.

For any total PS concentration ([PS]) and total FIXa concentration ([IXa_tot_], a given estimate of the global parameters *K_1_*, *K_2_*, *n_1_* and *n_2_* will define *P_0_*, *P_1_*, and *P_2_* allows, along with the assumptions enumerated above, calculation of F, Θ_222_/Θ_208_, R, and ΔP using equations S8, S9, S12 and S14 in terms of the local extensive parameters *F_1_*, $\left(\frac{\theta_{222}}{\theta_{208}}\right)_{1}$, *k_cat,2_*, and *δ_2_*. There are no extensive parameters associated with the observable ΔP.

These calculated quantities are then compared to the observed quantities shown in Figures 1 and 2 to obtain the sum of the squares of residuals (χ^2^) for each set of global and local parameters. $[FIXa],[FIXaPS^{1}],[PS^{2}FIXa], [PS^{2}FIXaPS^{1}]$ $[PS]$MATLAB, version R2011b (7.13.0.564; Mathworks, Inc, Natick, MA) was used to minimize χ^2^ so as to obtain estimates of all the parameters *K_1_*, *K_2_*, *n_1_*, *n_2_* *F_1_*, $\left(\frac{\theta_{222}}{\theta_{208}}\right)_{1}$, *k_cat,2_*, and *δ_2_*. $[FIXa_{tot}]=[FIXa]+[FIXaPS^{1}]+[PS^{2}FIXa]+[PS^{2}FIXaPS^{1}]$

These predicted observables were compared to the experimental data to calculate the sum of the squares of deviations of calculated values from experimental data (χ^2^). We minimized this with respect to the unknown dissociation constants and stoichiometries to obtain estimates of these parameters that allow us to describe all five data sets simultaneously (*i.e.*, globally). Dividing the minimized χ^2^ by the number of degrees of freedom (#data points - #parameters -1) yields the reduced chi squared ($\overline{\chi^{2}}$). If the number of data points is much larger than the number of fitting parameters, $\overline{\chi^{2}}$ should be close to 1 for a model that provides an adequate description of the data. For simplicity, all observables were expressed as ratios of values in the presence of C6PS to those for free FIXa, *i.e.*, the fluorescence and ellipticity recorded at the beginning of each experiment. Best fits with different assumed stoichiometries n_1_ and n_2_ are summarized in Table 2.
